# Functional Metabolic Diversity of Bacterioplankton in Maritime Antarctic Lakes

**DOI:** 10.3390/microorganisms9102077

**Published:** 2021-10-01

**Authors:** Antonio Picazo, Juan Antonio Villaescusa, Carlos Rochera, Javier Miralles-Lorenzo, Antonio Quesada, Antonio Camacho

**Affiliations:** 1 Cavanilles Institute for Biodiversity and Evolutionary Biology, University of Valencia, Paterna, E-46980 Valencia, Spain; antonio.picazo-mozo@uv.es (A.P.); juan.villaescusa@uv.es (J.A.V.); carlos.rochera@uv.es (C.R.); Javier.Miralles-Lorenzo@uv.es (J.M.-L.); 2 Departamento de Biología, Facultad de Ciencias, Universidad Autónoma de Madrid, E-28049 Madrid, Spain; antonio.quesada@uam.es

**Keywords:** next-generation sequencing, metabolism inference, functional diversity, microbial co-occurrence network, Byers Peninsula, maritime Antarctic lakes, FAPROTAX, biogeochemical cycles, Biolog Ecoplates

## Abstract

A summer survey was conducted on the bacterioplankton communities of seven lakes from Byers Peninsula (Maritime Antarctica), differing in trophic and morphological characteristics. Predictions of the metabolic capabilities of these communities were performed with FAPROTAX using 16S rRNA sequencing data. The versatility for metabolizing carbon sources was also assessed in three of the lakes using Biolog Ecoplates. Relevant differences among lakes and within lake depths were observed. A total of 23 metabolic activities associated to the main biogeochemical cycles were foreseen, namely, carbon (11), nitrogen (4), sulfur (5), iron (2), and hydrogen (1). The aerobic metabolisms dominated, although anaerobic respiration was also relevant near the lakes’ bottom as well as in shallow eutrophic lakes with higher nutrient and organic matter contents. Capacity for using carbon sources further than those derived from the fresh autochthonous primary production was detected. Clustering of the lakes based on metabolic capabilities of their microbial communities was determined by their trophic status, with functional diversity increasing with trophic status. Data were also examined using a co-occurrence network approach, indicating that the lakes and their catchments have to be perceived as connected and interacting macrosystems, where either stochastic or deterministic mechanisms for the assembling of communities may occur depending on the lake’s isolation. The hydrological processes within catchments and the potential metabolic plasticity of these biological communities must be considered for future climate scenarios in the region, which may extend the growing season and increase biomass circulation.

## 1. Introduction

The diversity and distribution of planktonic microbial assemblages in maritime Antarctic lakes have been widely studied during the last decades [1,2,3,4,5]. Maritime Antarctic lakes display a biological community mainly dominated by microorganisms [6,7,8,9,10], where bacterioplankton performs a key role [11]. The study of the structure and composition of the microbial community in aquatic ecosystems has been classically used as a first approach for their characterization. However, this type of approach may not be enough to understand how the composition of the microbial community affects ecosystem functioning [12,13]. The different microbial taxa present in an ecosystem can potentially display different metabolisms, therefore determining the functional profile of the microbial communities in an ecosystem. A variety of methods, based on the results obtained from the massive sequencing of 16S by next-generation targeted amplicon sequencing (NG-TAS), have been developed in order to infer community microbial functions. One of the most widely used methods in recent years has been FAPROTAX [14,15], which explores the main metabolisms of microorganisms associated with different biogeochemical cycles. Other alternatives include Phylogenetic Investigation of Communities by Reconstruction of Unobserved States (PICRUSt2) [16], Tax4Fun [17], BugBase [18], or IJSEM [19]. In the case of FAPROTAX, molecular data of the gene 16S rRNA sequences assigned to zero-radius operational taxonomic units (ZOTUs) are compared with a database where taxonomy is linked to the different potential metabolic functions related to the taxonomic assignation of each ZOTU, in order to obtain information on the functional metabolic patterns of the studied microbial community. This approach is applicable to a wide variety of environmental samples [14,20]. This type of functional inference accounts for potential activities, i.e., it is based on the presence of certain taxa with potential metabolic activities that may or may not ultimately be expressed in the ecosystem. Moreover, the metabolism is not exclusive; that is, a given taxon can potentially presents different types of metabolisms regarding the biogeochemical cycles of different chemical elements (C, N, S, Fe, H, etc.). Nevertheless, it is a very useful approach to understand the functional differences of microbial communities in aquatic ecosystems [21].

On the other hand, the possible interactions between the different microorganisms that form the microbial community have a direct effect on the assemblage of metabolisms expressed in these aquatic ecosystems. Thus, the study of the co-occurrence networks [22] of the different lakes can be useful to understand the differences in the type of dominant metabolisms and their intensity within the studied lakes.

The characterization of bacterioplankton metabolism, using bioinformatic tools, targeting the information included in available databases, represents a useful and fast way to provide knowledge about the role of a microbial community in the environment. However, these results alone do not provide true evidence of what is happening in the bacterial community, and further real physiological assays can be relevant to understand the relationship between potential and actual metabolisms. For this purpose, Biolog Ecoplates, including different types of carbon compounds, can be a reliable approach for the description of the preferences of the osmotrophic bacteria and its efficiency in using different organic sources involved in several metabolic pathways. The use of Biolog Ecoplates for the functional analysis of microbial communities through inoculation of natural samples of microorganisms was originally described by Garland and Mills [23]. This culture-based physiological approach has been recognized as a very effective tool to study the heterotrophic metabolism and to distinguish spatial and temporal functional changes in the microbial communities. This system, based on microwell plates containing commonly used microbial carbon sources, was originally optimized for the analysis of soil microbial communities [24]. However, the fact that most of their substrates are equally present in aquatic systems, especially carbohydrates and amino acids [25,26], make these plates a useful tool to evaluate the heterotrophic bacteria physiological diversity both in lakes [27] and glaciated habitats [28].

Current knowledge of aquatic ecosystems in Byers Peninsula, located in the South Shetland Islands (maritime Antarctica), is among the most complete and exhaustive for any place within the whole of Antarctica, as recently summarized by Rochera and Camacho [5]. In addition to all the information related to the global functioning of Byers’ aquatic ecosystems, the recent work that characterized the microbial community composition of Byers’ lakes through next-generation targeted amplicon sequencing (NG-TAS) [4] established the foundations for our functional study, that has been carried out with two different approaches, whose results are later compared. Primarily, the main potential metabolisms associated with the most relevant biogeochemical cycles (carbon, nitrogen, sulfur, iron, and hydrogen) were inferred from the taxonomic assignations obtained from the data of the gene 16S rRNA sequences assigned to ZOTUs. Later on, an approach to unveil the relative relevance of active carbon metabolisms in these ecosystems has been run for selected lakes, including depth profiles, using Biolog Ecoplates.

## 2. Materials and Methods

### 2.1. Study Site

Byers Peninsula is one of the largest ice-free areas in the Antarctic Peninsula region. It is located on the west side of Livingston Island, South Shetland Islands (62°04′35″ to 62°40′35″ S, 60°54′14″ to 61°13′07″ W). The site covers an ice-free area of around 60 km^2^ with a geomorphology mainly determined by periglacial landforms [29]. The milder weather in Byers Peninsula is typical of the maritime Antarctic region, which is in a clear contrast with the more severe climate conditions of the Continental region. It is characterized by cloudy skies, windy conditions, and relatively high precipitation, but with milder temperatures than in the Continental region. The minimum temperatures in winter average approximately −10 °C, and maxima are always below 0 °C. In contrast, summer temperatures are commonly above the freezing point [30,31]. Annual rainfall in the site is around 700 to 1000 mm [32].

In Byers Peninsula, there is a complex drainage network composed of permanent lakes, temporary shallow melt-water ponds, streams, and wetlands [33]. The lakes are distributed in two distinct geomorphological areas. Most studied lakes are located inland, on a plateau elevated around 100 m above sea level, whereas one of them (Lake Refugio) is located in the lowland platforms close to the beaches bordering the south coast (Table 1). Lakes located at different points of the central plateau (i.e., Chester, Midge, Escondido, Limnopolar, Turbio, and Somero) appear in sites modified by fluvial and periglacial processes that favor water retention. In general, these lakes show point surface inlets and outlets, except in the case of Lake Escondido, which is located in a hole between basaltic hills. In addition to surface feeding, there is also a groundwater flow over the permafrost towards the lakes [34], which is facilitated by the predominant sandy soils and gravels in the catchments. More information on the physical and chemical characteristics of the lakes can be found elsewhere [4,30,33,35].

The lake depth regulates the relative importance of the pelagic and benthic habitats, whereas the external nutrient inputs determine its trophic status [35]. The deeper inland lakes (<3 m) may show well-developed or patchy bottom moss carpets of *Drepanocladus longifolius*, however, these benthic mosses do not appear in the shallower lakes, which otherwise may show phototrophic microbial mats in their catchments or form a ring in the lake shore [5]. Among inland lakes, some catchments, such as those of lakes Chester and Midge, show a lower percentage of vegetation (both mosses and microbial mats) coverage compared to others, such as lakes Limnopolar and Somero. In general, lakes from the plateau are characterized by small watersheds and ultra-oligotrophic conditions [4]. However, a higher trophic status can be observed in sites such as Lake Turbio and, particularly, in the shallow Lake Somero, where a strong sediment–water interaction occurs due to the wave-induced re-suspension of sediments [35,36]. In clear contrast with those located in the inland plateau, the coastal Lake Refugio is located in an area showing a higher vegetation coverage, which is mainly composed of moss cushions. The beaches where this lake is located are also used by seals, mainly elephant seals [37], as a place to rest, and orthogenic soils also occur, particularly in coastal areas [38,39]. In contrast with inland lakes, Lake Refugio displays eutrophic conditions and a slightly higher salt content, mainly due to the influence of marine animals and sea spray, respectively [35,36].

### 2.2. FAPROTAX Analisys, Functional Prediction, and Metabolic Indices

The Functional Annotation of Prokaryotic Taxa (FAPROTAX version 1.2 October 2019, including updated taxonomies to be consistent with the SILVA release 132 database) was used to predict the functional potential of microbial communities [14,15] in the seven lakes studied, with two of them (lakes Chester and Limnopolar) including two samples each, one from surface waters and one from bottom waters. The complete database for FAPROTAX includes 7830 annotations and covers over 4722 taxa, being freely available at http://www.loucalab.com/archive/FAPROTAX/ (accessed on 2 May 2021). Our paired-end Illumina sequence data used in this study are available in the Sequence Read Archive (SRA) of the National Center for Biotechnology Information (NCBI), BioProject accession number PRJNA528697. Sequences from Illumina MiSeq system (2 × 250 bp, region V4 of 16S rRNA) were clustered in a ZOTU table with the UPARSE pipeline, and associated taxonomy were assigned with SILVA (release 132) and normalized by rarefying the reads of all samples to the minimum threshold of 2000 reads/sample. The characterization of the microbial community structure along different lakes studied, as well as a more detailed methodological description of the sequence analyses, can be found in [4].

The FAPROTAX output table contains the metabolic assignments of 90 different types of metabolisms, and each ZOTU in the input table can be assigned to one metabolism, to several metabolisms, or to none of them. From the 90 considered metabolism types, 28 include assignments of other metabolisms, which means that the raw output of FAPROTAX presents multiple duplicities in the metabolic assignments. For example, the metabolic assignment of nitrate respiration is the addition of three different assignments, all of them linked to anaerobic processes, such as, e.g., denitrification. The raw output of FAPROTAX presents, therefore, the same metabolic assignments in different metabolisms; for this reason, in our work, the FAPROTAX table has been debugged by eliminating duplicities in metabolic assignments. In this way, is the so-called “aerobic chemo-heterotrophy” by the FAPROTAX database will be referred to here as “Chemo-1”, and accounts for the potential heterotrophic use of carbon compounds other than biopolymers (i.e., lignin, chitin, xylan, and cellulose), one-carbon molecules (i.e., methanol and methane), and aromatic hydrocarbons. On the other hand, what is referred to in the FAPROTAX database as “aromatic hydrocarbon degradation” has here been grouped as “Chemo-2”. Finally, what is referred to as “chemo-heterotrophy” in FAPROTAX will be referred to here as “Chemo-3”, and it includes heterotrophic carbon metabolisms other than what we refer to as “Chemo-1” and “Chemo-2”.With regards to nitrogen, the three metabolic types in the FAPROTAX database called “nitrate denitrification”, “nitrate respiration”, and “nitrate reduction”, are all grouped in our work as “dissimilatory nitrate reduction”. Accordingly, there is correspondence between the original taxonomic reads of the ZOTU table without duplicities in the metabolic assignments generated by FAPROTAX. All analyses derived from metabolic data analyzed by FAPROTAX have been consequently performed from the modified table without duplicity in the metabolic assignment for nested FAPROTAX categories.

The Shannon–Weaver index (*H*), Shannon’s evenness index (*J*), Menhinick, and Chao1 indices were calculated for each sample using the metabolic assignments of the ZOTUs in the R Vegan package [40]. The Shannon–Weaver index [41] is used as a metabolic diversity index. Shannon’s evenness (*J*) is derived from the Shannon–Weaver index (*H*), and it is the ratio between the actual metabolic diversity of the sample and the maximum metabolic diversity which occurs when all the metabolisms are equally abundant. Menhinick’s richness index [42] is the ratio of the number of metabolic assignments to the square root of the sample size. Chao1 [43] is a nonparametric estimator of the minimum wealth (number of metabolic assignment) within a sample.

The “metabolic ratio” of the number and relative abundance (number of normalized reads) of the main metabolisms associated with the biogeochemical cycles of carbon, nitrogen, and sulfur, “C/N/S”, was calculated from the FAPROTAX metabolic assignment table without duplicity. Of the 90 metabolisms, 49 are metabolisms mainly associated to carbon, 16 to nitrogen, and 11 to sulfur. Thus, the metabolic ratio C/N/S was performed based on the addition of the metabolic assignments for each group and normalizing it to sulfur as 1.

### 2.3. Community Level Physiological Profiling

In order to evaluate the use of certain compounds that could be related to the predicted metabolic activities, an experimental analysis was developed for three of the selected lakes (Limnopolar, Somero, and Refugio), which, according to previous studies [35,36], are distributed along a trophic gradient from the oligotrophic Lake Limnopolar (including both surface and bottom samples for this lake), the mesotrophic Lake Somero, and the eutrophic Lake Refugio. Three of the seven lakes studied were selected for this assay, covering the most representative lake types along the trophic gradient, from oligotrophic (Lake Limnopolar), to mesotrophic (Lake Somero), and eutrophic (Lake Refugio). In this analysis, we used the multi-well Biolog Ecoplates system specifically designed for the study of environmental microbial community. These plates hold 31 different types (3 replicates each) of commonly used carbon sources, including 5 main groups of substances (amino acids, amines, carbohydrates, carboxylic acids, and polymers).

Biolog Ecoplates were inoculated with water of the assayed lakes and incubated for 12 days in the Byers camp laboratory, at a stable temperature of around 5 °C, similar to average in situ temperatures during summer. The detection of the metabolic activity was measured by the appearance of a purple color on the plates. This reaction occurs when growing microorganisms are able to degrade each carbon source, reducing the tetrazolium chromophore molecule bound to the carbon source, with the appearance of a purple color. This means that the assayed carbon compounds giving positive results can each be used as a main source of carbon to support bacterial growth of members of the community.

The metabolic activity in each plate was recorded every 24 h using high-resolution images taken by a camera, Canon EOS 350D (EF-S 18–55 mm, 1:3.5–5.6 DC) (Canon Inc., Ōta, Tokyo, Japan). Thereafter, images were analyzed and processed by image analysis software (ImageJ) to determine the intensity of the color developed in each well, which can be directly related to the intensity of the metabolic activity of the studied substances.

The intensity of the metabolic signal (bacterial growth on the substrate) in each plate was quantified using a qualitative scale from 0 to 3. For each positive carbon treatment, an average value from their three replicates was obtained. Treatments where the color signal only appeared in one of the three replicates were considered as false positives. The average ranked values, with their respective standard deviation, were used as an indicator of metabolic activity for each substance. Moreover, each carbon compound tested was clustered into one of the five different following groups to facilitate the data analysis: amino acids, carbohydrates, carboxylic acids, polymers, and amines [44].

To compare the intensity of the bacterioplankton metabolic activity of the selected lakes, the Average Well Color Development (*AWCD*) index suggested in [23] was used. This index serves as an approach of the metabolic activity in each lake.

$$AWCD=\sum \;\frac{\left(n_{i}-c\right)}{31}$$

*n_i_*: average qualitative color value of the three wells,
*c*: average qualitative color value of control wells.

Additionally, the Shannon–Weaver index (*H*) and evenness (*J*) indices were calculated to compare the metabolic diversity and evenness of each of the studied lakes. Both were derived from the measured qualitative color values in Biolog Ecoplates and quantified by the following equations:

H=−∑pilnpi

*pi*: ratio between *n_i_* (see *AWCD*) and the sum of *n_i_* of all other wells in the Biolog Ecoplates, and:

$$J=\frac{H}{lnS}$$

*S*: number of used carbon substrates.

### 2.4. Clustering and Multivariate Ordination Analyses

Cluster analyses (Heatmap) of the metabolic FAPROTAX assignment were performed separately; on one hand, for surface waters of the seven studied lakes, and on the other hand, for surface and bottom water of lakes Limnopolar and Chester. In order to understand and characterize the distribution of the different metabolisms inferred by FAPROTAX with respect to the use of the different carbon sources analyzed by Biolog Ecoplates, distance-based redundancy analyses (db−RDA) were performed based on Euclidean distances [45]. The analysis was carried out using the “pheatmap” package (version 1.0.12) and distance-based redundancy analysis (db−RDA) was performed with the statistical package Vegan, in the R programming environment [40].

### 2.5. Co-Occurrence Network Analysis

An inference of co-occurrence networks was performed from the sequencing data by the CoNet method [46] in the Cytoscape platform [47]. To avoid the compositionality bias, the relationships between bacterial compositional abundance were revealed by the combination of different metrics, correlations, and similarity/dissimilarity metrics, as detailed hereafter.

The network was constructed using the rarefied ZOTU table reported by Picazo et al. [4]. Each ZOTU was assigned to a specific lake if its abundance in that lake covered more than 90% of the total number of this ZOTU found among the studied lakes. Contrarily, ZOTUS where less than 70% was found in a single lake were classified as N.A. (ZOTUs not assigned to a lake). Network inference for surface water prokaryotic communities was performed with the CoNet method in the platform Cytoscape. We only considered the ZOTUs with a minimum total abundance of more than 10 reads for the sum of all lakes. The interactions between the nodes were then inferred using the following metrics: Pearson correlation, Spearman correlation, mutual information distance, Bray–Curtis dissimilarity, and Kullback–Leiber dissimilarity. A total of 1000 top and bottom edges were considered. The significance of the edges was assessed through a combination of permutation and bootstrap distributions generated with 100 iterations and enabling renormalization to avoid the compositionality bias. The edge final significance was obtained by merging the edge *p*-value (significance 5%) of each metric with Brown’s method, with a multiple testing correction through the Benjamini–Hochberg method. The final network was then visualized using the yfiles organic layout in the Cytoscape network visualizing software [47].

## 3. Results

### 3.1. Functional Structure of Bacterioplankton Communities

**Figure 1:**
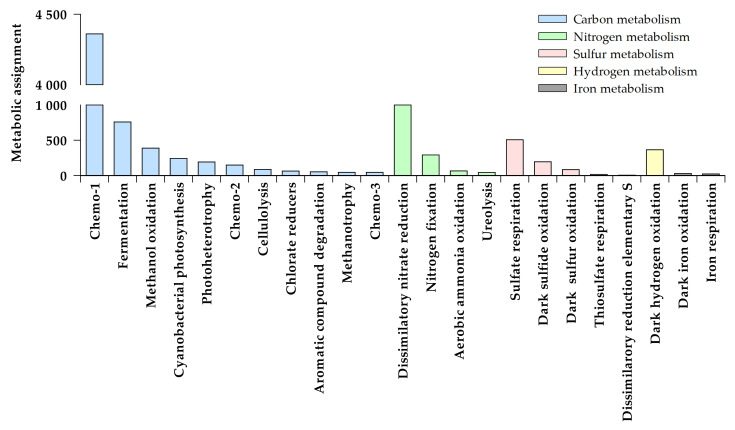
Abundance (metabolic assignments, number of associated reads) of the main metabolisms associated with the biogeochemical cycles of carbon, nitrogen, sulfur, hydrogen, and iron found in the whole set of lake samples.

A total of 23 main metabolism types were detected in the studied lakes based on the predictions made by FAPROTAX analyses (Figure 1). These functions were assigned over 607 ZOTUs of the 864 provided from the NGS ZOTUs table. The highest number of different potential metabolisms, 20, were predicted in the coastal eutrophic Lake Refugio, whereas they ranged between 6 and 15 in the lakes from the plateau. In those lakes where surface and bottom samples were analyzed (lakes Chester and Limnopolar), the number of predicted functions was slightly higher in the bottom samples. With regards to the relationship of these potential metabolisms with the biogeochemical cycles (Figure 1), a total of 11 and 4 were associated to the carbon and nitrogen cycles respectively, whereas other metabolisms were directly related to the sulfur (5), iron (2), and hydrogen (1) cycles.

**Figure 2:**
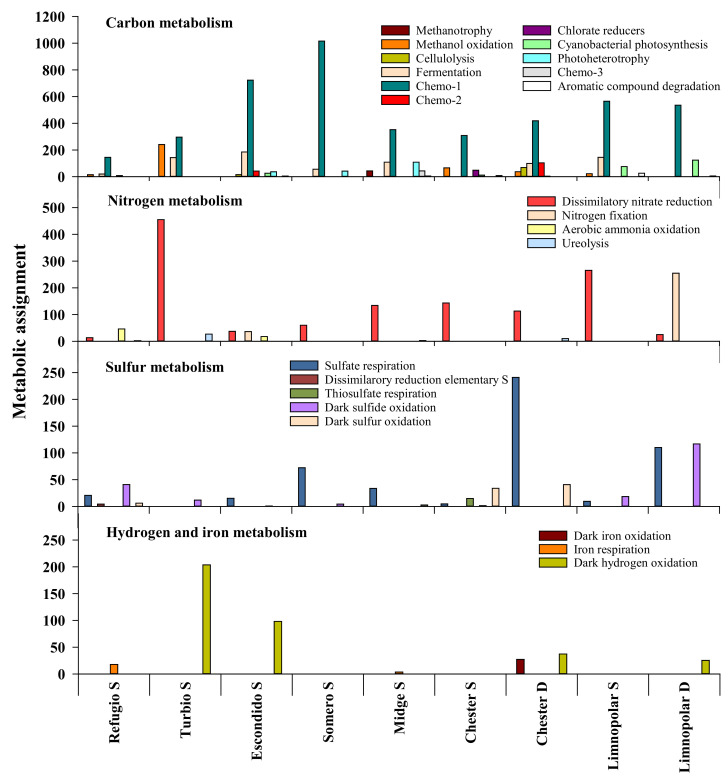
Main metabolisms associated with the biogeochemical cycles of carbon, nitrogen, sulfur, hydrogen, and iron in the different lakes studied in the Byers Peninsula, Antarctica. S stands for the surface sample, and D for the bottom (deep) sample.

Based on the reads obtained from the NGS analysis, the heterotrophic metabolisms Chemo-1 and fermentation were the most relevant metabolic functions associated with the carbon cycle in the studied lakes (Figure 1). The potential capability for degrading one-carbon molecules (i.e., methanol, methane), aromatic compounds, and some biopolymers such as cellulose, that we included in the so-called metabolisms “Chemo-2” or “Chemo-3”, were also detected in the studied lakes. In the case of nitrogen, dissimilatory nitrate reduction was the most commonly observed related metabolism (Figure 2), although other metabolic activities associated with the nitrogen cycle were also important in some of the lakes, such as nitrogen fixation. Concerning the sulfur cycle, both aerobic and anaerobic metabolisms such as the sulfide oxidation and sulfate respiration appeared as prominent.

#### 3.1.1. Heterotrophic Metabolisms

**Figure 3:**
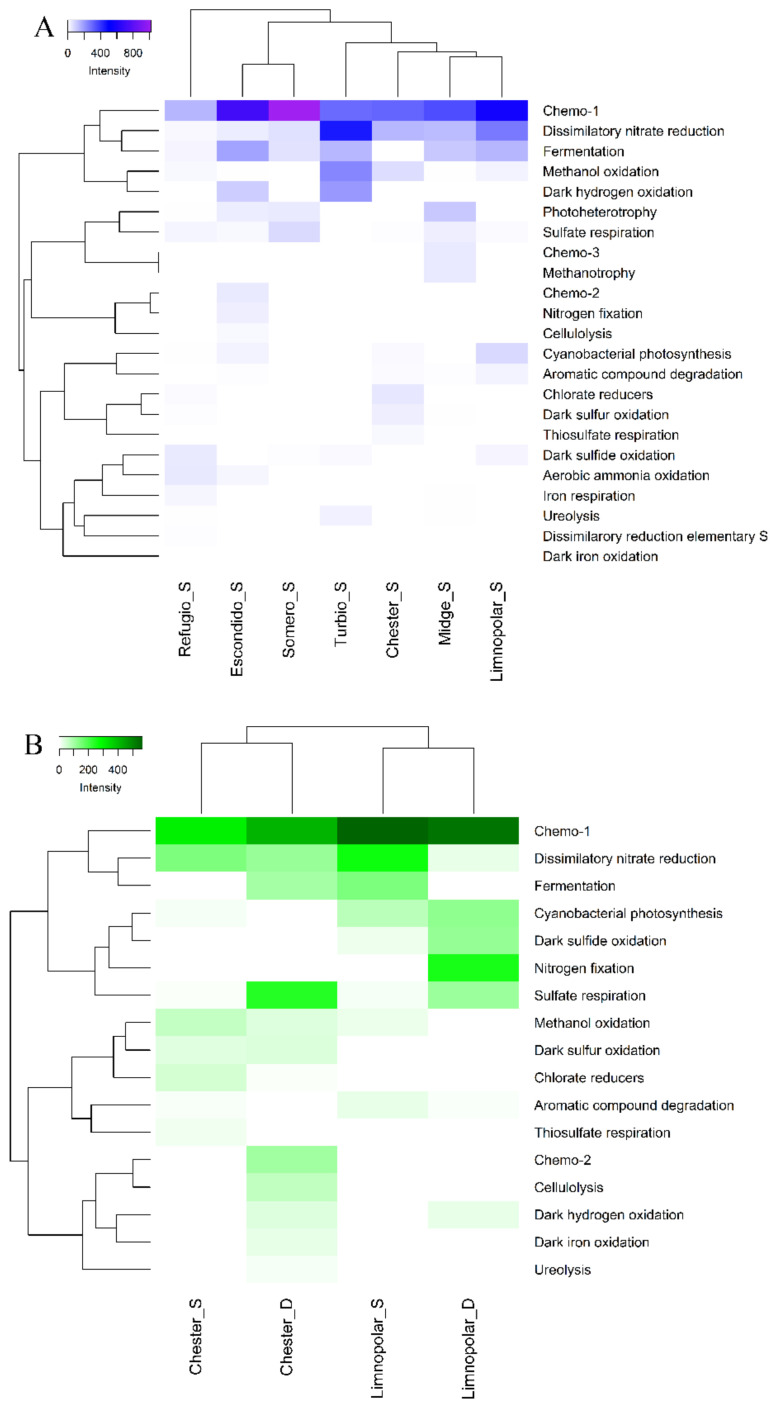
(**A**) Two-way cluster analysis (Heatmap) performed on the metabolic inferred FAPROTAX metabolisms in the surface waters of the studied lakes. (**B**) Two-way cluster analysis (Heatmap) performed on the metabolic inferred FAPROTAX metabolisms in the surface (S) and bottom (D) waters of the lakes Chester and Limnopolar. Samples were clustered using Bray–Curtis dissimilarities. The color intensity in the cluster dendrogram corresponds to the abundance (metabolic assignments) of normalized ZOTUs reads.

The capability for the Chemo-1 heterotrophic metabolism, that excludes the degradation of biopolymers, one-carbon compounds, and aromatic hydrocarbons from the considered heterotrophic metabolisms, was detected in all lakes and clearly dominated as the main potential metabolic pathway to obtain carbon and energy (Figure 2). In the lakes vertically profiled, this metabolism showed a roughly homogeneous distribution with depth (Figure 3). Chemo-1 metabolism was associated with 146 ZOTUs, among which Proteobacterial families dominated, such as *Sphingomonadaceae*, *Burkholderiaceae,* and *Pseudomonadaceae*. Capability for fermentation was still prominent, though less important (Figure 2); though widely distributed in all the lakes, it was only assigned to 28 ZOTUs. *Gammaproteobacteria* and *Bacteroidia* were the classes that more ZOTUs provided to this functional group. The Chemo-2 and Chemo-3 potential metabolisms were also detected in some of the lakes, although with much lower relevance (Figure 2). These hydrolytic activities were associated with the presence of the *Actinobacteria* and *Gammaproteobacteria*, of the genera *Gordonia* and *Acinetobacter,* respectively. Regarding the potential metabolism related to the hydrolysis of biopolymers, the capacity to hydrolyze cellulose was only detected in lakes Chester and Escondido (Figure 2). The associated ZOTUs varied among both lakes, with the *Bacteroidetes* genus *Lewinella* representing this potential activity in the lake Chester, whereas genera *Dyadobacter* and *Acidothermus* were those linked to this potential activity in Lake Escondido. In contrast, the potential ability to degrade other conventional biopolymers such as xylan and lignin was not detected in lakes.

Bacteria potentially able to obtain carbon for growth from one-carbon compounds were also present in the studied samples (Figure 2). Thus, a potential methylotrophic capacity was predicted in all the studied lakes, except in Lake Somero, representing a total of seven ZOTUs. Among them, those capable of oxidizing methanol clearly dominated over methane oxidizers. With respect to the rest of the lakes, the highest methanol oxidation capacity was found in Lake Turbio, particularly by the occurrence of the Alphaproteobacterial genus *Paracoccus*, whereas the lowest was observed in the surface waters of the deepest ultraoligotrophic Midge Lake. In this lake, methane oxidizers indeed showed a slightly greater potential relevance, being associated to the occurrence of members of the family *Methylomonaceae*. On the other hand, potential photoheterotrophs were also present in Midge Lake, as well as in Lake Escondido (genus *Rhodoferax*). This genus also appeared in shallower lakes such as Somero and Refugio, but with a lower abundance (Figure 2).

#### 3.1.2. Autotrophic Metabolisms

It should be noted here that photo-autotrophy refers only to cyanobacteria since the analysis with the 16S rRNA gene sequences attains only prokaryotes. Reads assigned to cyanobacterial photo-autotrophy distributed in nine ZOTUs and were more relevant in deeper lakes (<3 m), except in Lake Turbio (Figure 2). The occurrence of the *Phormidiaceae* genus *Tychonema* was spread among the studied lakes. In contrast, filamentous forms of *Leptolyngbya* and *Nostoc* showed a more restricted distribution, but were also relatively abundant. It is noteworthy that these cyanobacteria are widely distributed in the microbial mats growing on lake catchments, so that they could have an allochthonous origin. Among truly planktonic picocyanobacterial forms, the genus *Cyanobium* occurred only in lakes Limnopolar and Escondido.

The potential capability for chemolithoautotrophy also appeared in the studied lakes. Competence for sulfide oxidation potentially occurred in all the lakes, and more particularly associated with the bottom of Lake Limnopolar (Figure 2 and Figure 3). Two *Gammaproteobacteria* genera, *Thiobacillus* (8 ZOTUs) and *Thiothrix* (1 ZOTU), were the most commonly found with this metabolic function. Chemolithoautotrophic ammonia oxidizers of the family *Nitrosomonadaceae*, responsible for nitrification, occurred particularly in the ammonium-rich eutrophic Lake Refugio (Figure 2), and were represented by a total of 10 ZOTUs. Likely, nitrification was also predicted in Lake Escondido by the presence of one of the scarce archaean ZOTUs observed, corresponding to the family *Nitrososphaeraceae*.

#### 3.1.3. Other Respiratory Metabolisms

Microbial taxa, reportedly able to perform the dissimilatory nitrate reduction, appeared in all the studied lakes, but they were relatively more relevant in the surface waters of the deeper and more oligotrophic lakes, such as Chester, Midge, and Limnopolar (Figure 2 and Figure 3). The eight ZOTUs involved belong to the phylum Proteobacteria, with *Stenotrophomonas* being the most important genus among them. Capability for the dissimilatory nitrate reduction was also relevant in Lake Turbio, but in this case mainly associated with the occurrence of genus *Paracoccus*. Hydrogen oxidation was also predicted to occur by FAPROTAX, also being linked to the above-mentioned genus and lake.

Other potential metabolisms related to the respiration of inorganic compounds also appeared in the studied lakes. Metabolic capacity for respiration of sulfur compounds was also detected in most of the lakes, with sulfate respiration being the most widespread and dominant (Figure 2), particularly in the deep waters of the vertically profiled lakes (i.e., lakes Chester and Limnopolar), being mostly associated to the presence of the family *Desulfobacteraceae*. On the other hand, the potential for dissimilatory reduction of elementary sulfur, in this case associated to genus *Desulfuromonas*, was only detected in the eutrophic Lake Refugio, which was also potentially responsible of the iron respiration in this lake jointly with *Geobacter*. On the other hand, potential capacity for thiosulfate respiration was only detected in Lake Chester, linked to the occurrence of the Gammaproteobacterial genus *Alishewanella*. Concerning other less common respiratory metabolisms, chlorate-reducing bacteria of genus *Dechloromonas* were also present in lakes Chester and Turbio, being more abundant in the former. Finally, 8 ZOTUs of hydrogen-oxidizing bacteria, all belonging to genus *Hydrogenophaga*, appeared in Lake Turbio.

#### 3.1.4. Assimilation of Nitrogen Compounds

Nitrogen fixation capability was not detected in all the studied lakes, but it was, instead, especially important in the bottom of Lake Limnopolar (Figure 3), associated with the presence of genus *Bradyrhizobium*. Hydrolysis of urea was also predicted in some of the lakes, with more reads occurring in Lake Turbio compared to the others. In this case, this was associated with the presence of the *Planctomycetes* genus *Singulisphaera*, whereas in other lakes, this metabolic capability was related to the occurrence of Proteobacterial genera such as *Roseomonas* or *Methylophilus*.

### 3.2. Carbon-Substrate Utilization Profiles

**Figure 4:**
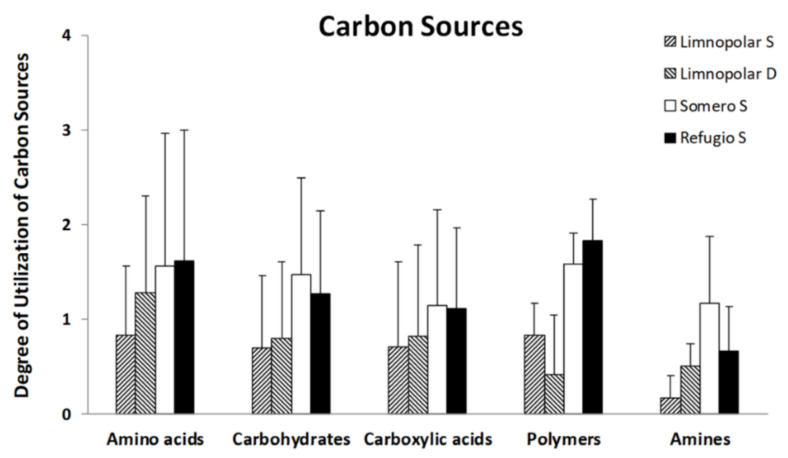
Average utilization values for the different carbon source groups (Biolog Ecoplates) by the microbial community of the Lake Limnopolar (S = surface and D = Deepest waters) and that of the surface water of the shallow lakes Somero and Refugio.

The results of the utilization of commonly used carbon sources from Biolog Ecoplates (grouped by types) for each selected lake within a trophic gradient are shown in Figure 4. Among the three studied lakes, the most used carbon source types were, on average, amino acids (AA) and polymers (P), both in the eutrophic Lake Refugio, followed by carbohydrates (C) and carboxylic acids (CA). Amines (A) displayed the lowest utilization degree, however, in this case, for the mesotrophic Lake Somero, the values were higher compared to Lake Refugio. The calculated AWCD index showed values of 0.71 and 0.83 for the oligotrophic Lake Limnopolar surface (S) and bottom (D) respectively, 1.39 for Lake Somero, and 1.32 for Lake Refugio, showing an increase in the metabolic activity in the systems from oligotrophic conditions to a higher trophic status.

**Figure 5:**
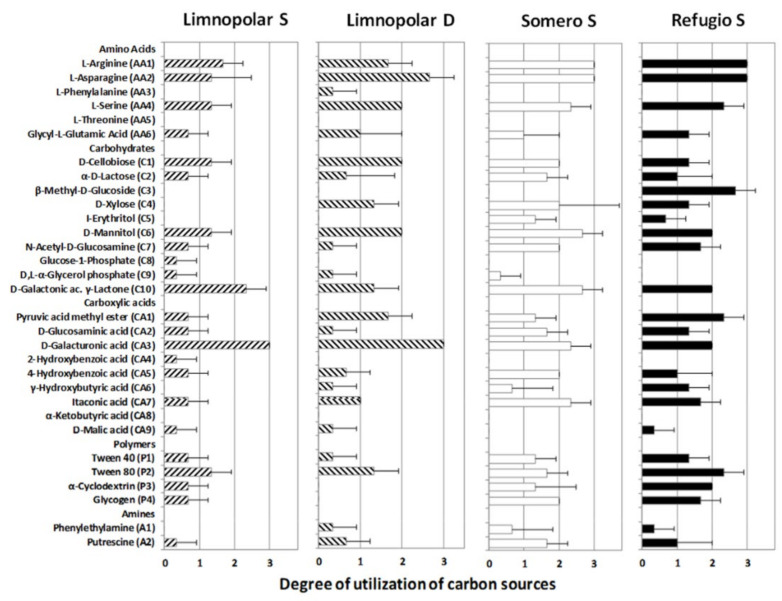
Carbon sources’ average utilization profiles (Biolog Ecoplates) for samples of Lake Limnopolar (S = surface and D = Deep waters), and surface waters of the shallow lakes Somero and Refugio.

Distinctive patterns for metabolizing organic carbon compounds emerged in lakes when Biolog Ecoplates profiles were compared (Figure 5). For amino acids (AA), the highest values appeared in Lakes Refugio and Somero. It is noteworthy that these lakes displayed high utilization values for compounds such as L-arginine (AA1) and L-asparagine (AA2), that are very common in nature and are usually metabolized by microorganisms. Most of the assessed carbohydrates were easily used in all lakes, though β-methyl-glucoside (C3), which is a monosaccharide derived from glucose, was slightly metabolized in lake Refugio. Additionally, phosphorylated compounds such as glucose-1-phosphate (C8) and glycerol-phosphate (C9) showed very low utilization.

The utilization of carboxylic acids showed a similar pattern to that observed for amino acids and carbohydrates, however the differences among the studied lakes were lower than the previously mentioned compounds. Lakes Refugio and Somero also showed the highest consumption rates in this case. Most carboxylic acids were easily used, although some compounds such as 2-hydroxybenzoic acid (CA4) or α-ketobutyric acid (CA8) were hardly metabolized. It is very noticeable that, unlike the rest of the compounds, D-galacturonic acid (CA3) was more efficiently metabolized in Lake Limnopolar. Additionally, the physiological activity over polymeric compounds showed a similar pattern to the previously mentioned organic compounds. Among polymeric compounds, Tween−80 (P2), which is a commercial derivate of oleic acid, showed the highest utilization values for all tested lakes. Finally, the metabolizing capability over amines showed a slightly different pattern compared to all those observed previously, since Lake Refugio did not show the highest values. The higher utilization values in Lake Somero were given by the utilization of putrescin (A2), which is a compound related to the breakdown of amino acids.

### 3.3. Statistical Analyses and Diversity Parameters

The statistical and diversity parameters obtained from both the functional annotation and the carbon-substrate utilization profile studies are summarized in Table 2. With regards to the functional annotation performed with FAPROTAX, the Chao-1 values ranged from 6 to 20, being higher in the eutrophic coastal lake Refugio. The Shannon–Weaver index (*H*) also reached the highest values (~2) in Lake Refugio, although differences with inland lakes were not so marked. The relative importance of metabolic activities based on the element involved are expressed *ad hoc* as the metabolic C:N:S ratios, which are the quotients between the numbers of predicted functions related to each element. The ratios indicated a higher incidence of carbon-related metabolisms than those of nitrogen and sulfur. In general, these ratios were more balanced (that is, lower dominance of C-associated metabolisms) with increasing depth and trophic status.

With regards to the outcomes of Biolog Ecoplates analyses, the Shannon–Weaver index (*H*) showed values of 2.93 and 2.89 for Lake Limnopolar S and D respectively, 3.09 for Lake Somero, and 3.12 for Lake Refugio. Thus, the highest metabolic diversity values appeared in lakes Somero and Refugio, both displaying a higher trophic status. The equitability evenness index (*J*) showed values of 0.93 and 0.92 for lake Limnopolar S and D respectively, 0.99 for Lake Somero, and 0.97 for Lake Refugio.

**Figure 6:**
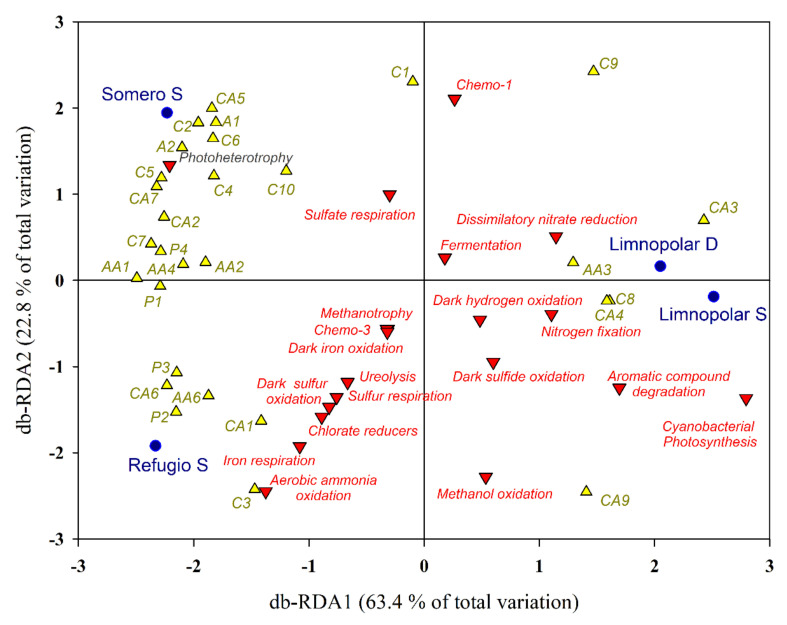
Distance-based redundancy analysis (db−RDA) tri−plot between the metabolic inferred FAPROTAX metabolisms and the results of the most commonly used carbon sources (codes for Biolog Ecoplates as in Figure 5) for lake Limnopolar (S = surface and D = deep waters) and surface waters of lakes Somero and Refugio.

The axes of the db−RDA performed with data of carbon utilization and the predicted functions explained 63.4% and 22.8% of the total variance observed (Figure 6). The first axis clearly discriminated the oligotrophic Lake Limnopolar from the mesotrophic and eutrophic lakes Somero and Refugio, respectively. Both sampled depths for Lake Limnopolar were situated on the positive side of the first axis, which was more related with the occurrence of cyanobacterial photosynthesis, nitrogen fixation, and the potential use of aromatic compounds. In contrast, lakes Somero and Refugio were positioned on the negative side and were related with a higher metabolic potential to use polymers and most of the amino acids. The second axis discriminated on the positive and negative sides the lakes Somero and Refugio, respectively. Dissemination of lakes within this axis was related to a higher occurrence of metabolisms involving electron acceptors other than molecular oxygen in Lake Refugio, and this lake has more eutrophic conditions with more anaerobic micro-niches.

### 3.4. Co-Occurrence Network of Bacterioplankton Communities

**Figure 7:**
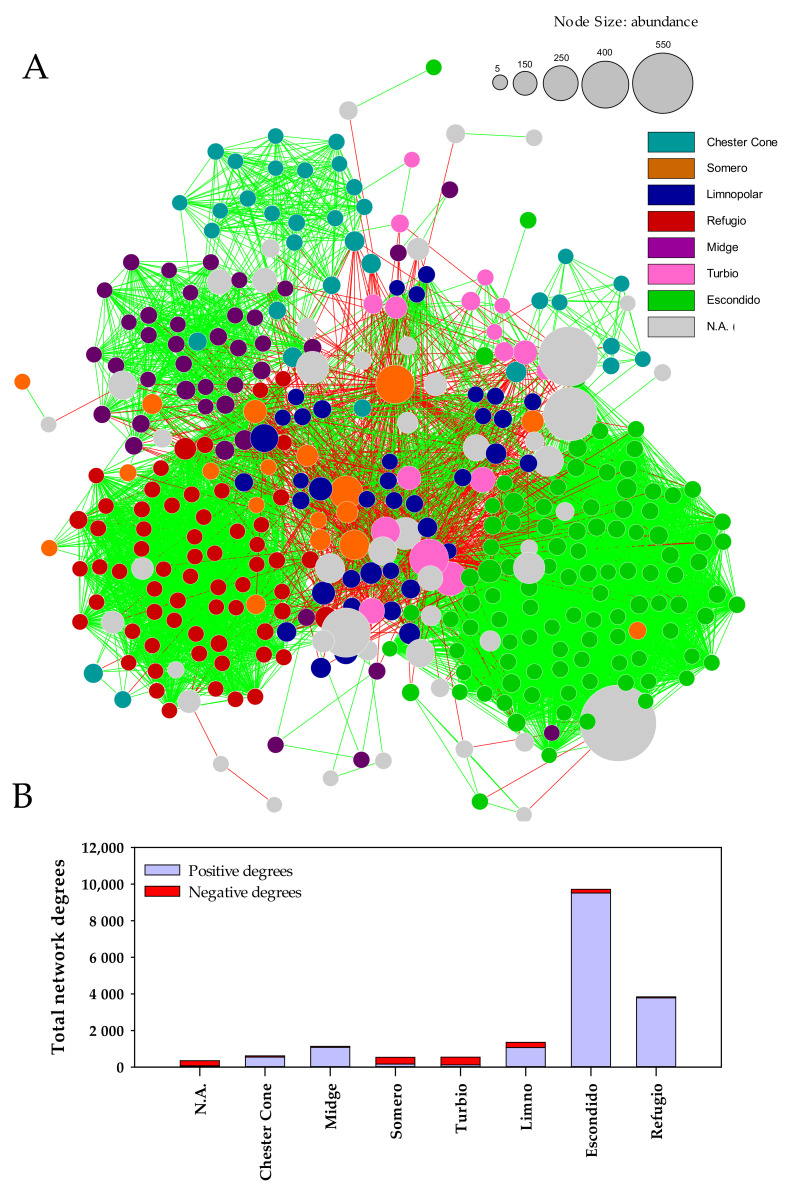
(**A**) Bacterioplankton co-occurrence network of the Antarctic lakes studied, based on the relative abundance matrix previously reported by Picazo et al. [4]. The nodes represent ZOTUs sequences. The size of the node indicates the relative (rarefied) abundance, and the color of the nodes indicates the level of specificity of ZOTUs for a certain lake, in such way that when at least 90% of reads are associated to a single lake, the node of the ZOTU shows the same color of the lake indicated in the legend for the lake. Nodes with significantly positive or negative co-occurrence for any of five measures used (Pearson correlation, Spearman correlation, Bray–Curtis dissimilarity, Kullback–Leiber dissimilarity, and mutual information similarity) are joined with a single edge. (**B**) Total network degrees obtained in the co-occurrence analysis for each of the studied lakes distinguishing the positive and negative relationships between nodes (ZOTUs). N.A. = ZOTU not assigned specifically to any lake.

A co-occurrence network was assembled based on the abundance ZOTUs matrix of the prokaryotic communities of the lakes (Figure 7A). Co-occurrence networks allow to establish the statistically significant relationships (edges) between the different taxa, ZOTUs (nodes) in this case, from five different metrics (Pearson correlation, Spearman correlation, mutual information distance, Bray–Curtis dissimilarity, and Kullback–Leiber dissimilarity). The unification of these metrics can be positive (green lines) if the ZOTUs appear co-occurring together, or negative (red lines) if the ZOTUs are mutually exclusive (when one appears in a lake, the others do not appear). The node size shows the relative abundance of each ZOTU. The color of the nodes indicates the level of specificity of ZOTUs for a certain lake, in such way that when at least 90% of reads are associated to a single lake, the node of the ZOTU shows the same color of the lake indicated in the legend for the lake. On the other hand, ZOTUs with a more spread distribution that do not meet this specificity threshold are referred to as N.A. (not assigned to a lake) nodes (grey color in Figure 7).

The network showed a high proportion (~89%) of ZOTUs being mostly associated (>90% of the detected reads for the ZOTU) to a particular lake, which highlights a high degree of specificity of part of the microbial community. Lakes Chester, Midge, Refugio, and Escondido were totally differentiated in the network, based on the composition of their bacterioplankton assemblages (Figure 7A). They constituted stable sub-networks, showing a gradient of complexity (i.e., degree of connections) from the lowest complexity in Lake Chester to the highest in Lake Escondido.

The sub-networks formed by the ZOTUs assigned to specific lakes have different network degrees, as well as a different ratio of positive and negative degrees. The total number of network degrees provides insight into the complexity of the network in that lake. If the degrees of these sub-networks are mainly positive, this is an indication that the network is stable and dominated by co-occurrence relationships. Contrarily, if negative degrees dominate, this means that what characterizes the specific ZOTUs of that lake is the exclusion with respect to the ZOTUs present in the other lakes, then they do not present a stable internal network of co-occurring taxa. 

Lakes Refugio and particularly Escondido displayed the highest number of relationships between nodes (degrees), mainly co-occurrence relationships (positive degrees) (Figure 7B). Chester Cone and Midge Lake showed a sharp decline in the complexity of their networks, although the sub-network they form is still dominated by positive relationships with stable co-occurrence connections between different taxa (Figure 7B).

Lake Limnopolar showed a similar number of degrees as Midge Lake and Chester Cone, which implies that this lake shows a relatively complex sub-network, and most of these connections (edges) are positive with co-occurring species (ZOTUs) with stable relationships. However, it is noteworthy that Lake Limnopolar has a relatively high number of negative relationships (Figure 7B), which implies that some of the taxa (ZOTUs) that form its sub-network have very strong exclusion relationships with the rest of the lakes.

Lakes Turbio and Somero were disposed in a weakly connected central sub-network and showed the lowest stability among the bacterioplankton assemblages of the studied lakes, with a low number of connection degrees that mainly showed exclusionary relationships (negative degrees).

## 4. Discussion

Our results provide both a functional annotation, conducted with data of sequencing of bacterial 16S rRNA genes previously obtained in some lakes from Byers Peninsula [4], and a community level physiological profile of the differential utilization of carbon sources by these microbial communities. This study represents a first attempt to prospect functional patterns of microbial consortia in lakes of the maritime Antarctica related to different environmental settings. Our findings show the occurrence of general metabolic functions, which had been previously reported to be common properties of microbial communities within diverse lake water environments [48,49,50,51]. Former limnological studies conducted in Byers Peninsula show the occurrence of a gradual nutrient enrichment in lakes as they are closer to the sea [33,35], which in part determines the structure of bacterioplankton communities [4,36]. In this sense, shallow coastal lakes such as Lake Refugio are eutrophic, mostly because of the nutrient inputs coming from marine animals resting there, whereas inland lakes are generally oligotrophic, with otherwise very limited inputs of nutrients. However, some shallow inland lakes, such as Lake Somero, are mesotrophic because of the occurrence of a biologically mediated active nutrient release from the sediments. The prediction of major functional roles obtained in our FAPROTAX study delivers a clustering of the lakes resembling that observed in the previous research studies.

As might be expected from the physical and chemical features of the lakes during the summer season, a prominent role of the aerobic metabolism in these bacterioplankton communities was found. Accordingly, vertical oxygen profiles in the deeper lakes of Byers are essentially orthograde [30], which agree with their oligotrophic conditions and the oxygen produced by the benthic flora. However, in some cases, oxygen may decrease with depth [33], particularly at the beginning of the summer ice-free period, thus suggesting an oxygen depletion during winter below the ice cap. In this sense, some of the lakes studied were partially ice-covered during the sampling survey [4]. This, together with the influence of sediments in samples obtained from the bottom of lakes, may explain the occurrence of microorganisms with potential anaerobic metabolism, such as the dissimilatory sulfate and nitrate respirations, as well as the occurrence of methane oxidizers, as in Midge Lake. However, the latter does not seem to be ecologically significant in these lakes given the virtual absence of methanogens. Despite the absence of summer stratification in Byers’ deepest lakes, there are studies demonstrating the differential vertical distribution of bacterioplankton assemblages with depth [36]. This functional contrast, which occurs despite the physical homogeneity of the water column allowed by the micro-niches favored by the presence of dense moss carpets, is well-illustrated here by the contrasting C:N:S metabolic ratios observed in the profiles of Lakes Limnopolar and Chester among the number of metabolisms related to the carbon, nitrogen, and sulfur cycles, respectively. These ratios clearly indicate an increased relative importance of N- and S-related dissimilatory respiratory metabolisms [52] in deep lake waters compared to C metabolisms in surface waters (Table 2).

Our results demonstrate that bioavailable nitrogen can also enter the lakes’ food webs through nitrogen fixation, which is a good strategy in environments where nutrients are externally supplied in very low amounts. Previous studies conducted in Byers already showed this diazotrophic capacity, but particularly in microbial mats thriving in the catchment of lakes [53]. Our study demonstrated that this also extends to within-lake communities, although some of the diazotrophic cyanobacterial sequences found in the plankton samples are also abundant in these mats and could reach the lake by runoff from the microbial mats within the lakes’ catchments. It is interesting to note that physiological studies performed in Byers with benthic communities show that this activity of diazotrophs is greatly regulated by temperature [54], whereas other metabolisms such as the uptake of nitrate and urea, also predicted by our functional analysis, appear to be less sensitive to this factor.

The carbon-substrate utilization profiles obtained with the Biolog Ecoplates provide the actual expressed metabolism in the lakes where these assays were performed (Limnopolar, both surface and bottom samples, Somero, and Refugio). The results show that amino acid metabolism is more important in lakes with higher trophic status, such as Refugio and Somero. Among them, compounds such as L–Arginine (AA1) and L-asparagine (AA3) represent a common source of carbon, nitrogen, and energy, which, in Antarctic freshwater ecosystems, is derived mainly from primary production [55]. Thus, these lakes display higher trophic status than other ultra-oligotrophic lakes such as Limnopolar, and consequently, the autochthonous productive processes are more relevant. Consumption of other amino acids, such as L-phenylalanine (AA3), appear mostly related to the bottom of Lake Limnopolar. This compound is used in the synthesis of flavonoids, which are involved in UV filtration, symbiotic nitrogen fixation, and pigmentation. Accordingly, the diazotrophic *Bradyrhizobium* mostly occurs in the bottom of Lake Limnopolar, where it could be associated with mosses covering the lake bottom [4]. The occurrence of these mosses increases the spatial heterogeneity between the surface and the bottom of the lake, which results in a differentiation of ecological niches and taxa, and therefore of associated metabolisms [56].

Simple carbohydrates, such as glucose, are the most common and easily metabolized form of organic carbon source for microorganisms. However, often, they are not available in their primary forms, and other products derived from degradation or decomposition are present. This is the case for β-methyl-glucoside (C3), which is a monosaccharide derivate from glucose by acid degradation and decomposition processes, that is especially relevant in lakes Refugio and Somero. Vegetation from the catchments, that is composed principally by microbial mats and terrestrial mosses, provides an important supply of organic carbon for these lakes, particularly during the onset of the summer season because of the intense run-off processes produced when the snow melts [5]. Accordingly, it is expected that there is a close link between these plant and algal materials and the metabolic capabilities of bacteria to degrade them. D–cellobiose (C1) is a carbohydrate produced by the hydrolysis of cellulose both under anoxic and oxic conditions in soils [57]. This compound represents one of the major sources of carbon for soil bacterial communities. Accordingly, our FAPROTAX analysis predicted the functional capability of these communities to degrade cellulose.

Among carboxylic acids, D-galacturonic acid (CA3) activity appeared in all the studied lakes, but it is more important in Lake Limnopolar. This compound is an oxidized monosaccharide form of D-galactose that is present in the mucilage produced by the cyanobacterial microbial mats [58]. Microbial mats cover part of the catchment of Lake Limnopolar [53] and constitute an important source of allochthonous carbon to the lakes [5]. D-malic acid (CA9) is a dicarboxylic acid which is produced by living organisms. Malates are used in the pyruvate/malate cycle that merges carbohydrates, lipid, and protein metabolisms. On the other hand, the capacity to degrade 2-hydroxybenzoic acid (CA4) seems to be more important in the surface of Lake Limnopolar. This is an aromatic compound usually derived from plants and related to their growth, development, photosynthesis, transpiration, iron uptake, and transport. The db–RDA (Figure 6) also relates the surface of Lake Limnopolar with aromatic compound degradation metabolisms, which is in accordance with these results.

The degradation of complex polymers such as Tween 80 (P2) and α-cyclodextrine (P3) requires a complex metabolic capability. The activity of these compounds is more important in Lakes Refugio and Somero, which display the higher trophic status and more diverse metabolic capabilities. The degradation of these compounds could be related to some forms of polymer degradation, such as cellulolysis, which was also predicted by our functional annotation performed with FAPROTAX. In this sense, the db–RDA shows that this cellulolytic activity is prominent in Lakes Refugio and Somero, supporting the results obtained from Biolog Ecoplates. On the other hand, some amines, such as putrescine (A2), are produced by the degradation of amino acids and are commonly found in lacustrine sediments. The extreme shallowness and the effect of the wind favor removal processes and increase the availability of this compound in the water column of shallow lakes such as Refugio and Somero, with animal supplies being an additional important enhancement factor in Lake Refugio.

The structure and function of the bacterioplankton communities inhabiting Byers lakes are sensitive to trophic conditions [4]. As a general rule, a positive relationship is expected between biodiversity and ecosystem function. Figure 7, where data of both the potential metabolisms and those of the Biolog Ecoplate metabolic profiles are jointly used, clearly shows a separate clustering of samples originating from oligotrophic (Limnopolar S and D) or from more eutrophic lakes (Somero and, especially, Refugio). The db–RDA (Figure 6) shows a first axis which separates the lakes on a trophic gradient, with the most oligotrophic sampling sites (Limnopolar S and D) set at one extreme of the gradient, and the more eutrophic (Somero and, especially, Refugio) on the opposite end. The second axis, however, discriminates among the last two lakes, which also show strong ecological differences mostly related to the predominant nutrient sources, mainly deriving from the internal load in Lake Somero, and from external supplies by marine resting animals in Lake Refugio.

A higher structural and functional diversity was detected under the more eutrophic conditions (i.e., Lake Refugio), as well as a more diverse metabolic capacity to exploit carbon sources. This is apparently inconsistent with the regularly observed pattern in temperate regions, consisting in higher values of diversity, both structural and functional, at intermediate trophic status [12]. However, in temperate lakes, the environmental constraints to both the structural and functional diversity are less relevant than in environments, such as the extreme Antarctic lakes, where the environmental constraints (low temperature, nutrient limitation, and others) would depress both taxonomic and functional diversity. In Antarctic lakes, the strong limitations posed by restricting physical and chemical conditions could be counteracted by the increasing availability of trophic resources at the upper part of the trophic gradient, and this would explain why diversity maximization at intermediate trophic levels, as it occurs in temperate lakes, would not be displayed by lakes in extreme environments, such as those studied here. These, instead, maximize their functional diversity as the higher trophic level increases the metabolic niches to be exploited, thus counteracting other environmental restrictions.

Environmental drivers other than trophic status may exist (e.g., sea influence, nearby birds and mammals, etc.), bringing external elements to the microbial community and, therefore, to their associated functions. These forcing factors appear to shape the co-occurrence network obtained with the ZOTUs. Topological features of this network reveal a spatial pattern showing most lakes with microbial communities being well-differentiated from each other. However, a high degree of specialization on environmental conditions explaining these findings could be complemented by the fact that the geographical location within the Peninsula and some characteristics of the catchment could make some lakes more isolated than the others, displaying differentially external influences (sea birds and other marine animals; vegetation, including microbial mats, in the catchments; sea spray, which would explain the spacing of the sub-networks observed in Figure 7). This is the case of Midge Lake and Chester, which are in an inner part of the plateau of the Peninsula, where external inputs are more limited. Furthermore, the catchment of these lakes, which are one near the other (Figure 1), is very scarce in vegetation coverage and benthic communities that might provide foreign microbiota and somewhat counteract the ultraoligotrophic characteristics of these lakes. This situation is even more marked in Lake Escondido, which is located in a land depression between basaltic hills [33]. However, because of the common ultra-oligotrophic character of these lakes, they still share some functional features of their microbial communities that differentiate them from other lakes of the site, as described previously.

This isolation is in contrast with that observed for lakes arranged in the central part of the network (i.e., Limnopolar, Somero, and Turbio), which are more exposed to both waterborne and airborne inputs. These lakes are furthermore surrounded by catchments in which benthic communities flourish (principally microbial mats), which are known to be a source of allochthonous microbiota, as observed in Lake Limnopolar ([5] and references cited therein). Another example of this would be the high occurrence of bacterial groups such as Flavobacteria populating Lake Somero, which are closely associated with the cyanobacterial mats growing nearby the lake [4]. Accordingly, network interactions in these lakes are likely stronger than those envisioned in this network, due to the occurrence of this high connection with the surrounding catchment that is not evaluated here. All this led to consider the possibility to perform a wider network meta-analysis including these benthic microbial communities to shed more light on these interactions between the catchments and the lakes.

Both lakes Refugio and Escondido showed a higher number of network degrees compared to the other sites, although they differ notably in their characteristics. A higher size of the Refugio sub-networks could be attributed to greater diversity occurring in this copiotrophic environment. Nevertheless, it is the more isolated and oligotrophic lake Escondido which shows a higher connection and positive associations within the lake. This appears to be contradictory with the idea that positive interactions increase under eutrophic conditions because of reduced competition for resources [59]. In this case, this could be explained by the occurrence of important dispersal barriers, as argued by other authors [60].

## 5. Conclusions

This is the first comprehensive study reporting the utilization of carbon sources, the functional annotation, and network association patterns of bacterioplankton communities in lakes from the maritime Antarctica. It has been successful in distinguishing these communities based on their capacity to use diverse types of organic matter sources, as well as providing general insights on lakes’ ecosystem functions and linking with the catchment. This has revealed a significant capacity to use diverse carbon sources other than labile forms freshly derived from primary production, which should have important consequences for the biogeochemical cycles in these aquatic environments. Differences observed between lakes support the idea that a high environmental heterogeneity occurs, in spite of the environmental uniformity a priori imposed by the extreme cold conditions. Likewise, depth variations in the structure and function of their bacterioplankton communities can be substantial and should be considered if the knowledge of the functioning of the whole system is addressed. The study also reveals the incidental importance that anaerobic and/or “dark” metabolisms may have during winter in these lakes, when they are covered by ice and snow, and then the light attenuation and oxygen depletion are maximized, even though there is a clear dominance of aerobic metabolisms during the studied summer ice-free period. However, although our FAPROTAX and Biolog Ecoplates analyses can be regarded as a useful screening to link structure and metabolic functions, further validation of our results with additional sound analyses is needed, by applying, for instance, a meta-transcriptomic approach. The co-occurrence association network produced with the taxonomic bacterial data indicated the markedly variable interactions among bacterial groups depending on the isolation of lakes related to their capacity to be affected by external factors. This provides insights into the potential consequences of the advent of wetter and warmer climatic scenarios envisioned for this region, where hydrological dynamics in the catchments, and thus connectivity, would likely notably increase. Additionally, a hypothetical extension of the growing season and the increase in nutrient availability would provide more opportunities for taking advantage of all the metabolic functions predicted by the survey, as it evidenced that a high trophic status did not necessarily decrease the functional diversity and network interactions in these cold environments.

## Figures and Tables

**Table 1:** Geographical location and main characteristics of the studied lakes from Byers Peninsula. In the analysis column, F stands for the analysis performed with FAPROTAX, whereas B stands for those performed with Biolog Ecoplates. The estimation of the trophic status of lakes is based in Rochera et al. [35].

Lake	X-UTM	Y-UTM	Catchment Size (km^2^)	Lake Surface (km^2^)	Maximum Depth (m)	Analyses Conducted	Trophic Status
Refugio	602200	3050550	0.12	0.016	0.5	F, B	Eutrophic
Turbio	598000	3051800	0.58	0.021	7.8	F	Oligotrophic
Escondido	599475	3052650	0.08	0.022	4.5	F	Ultra-oligotrophic
Somero	596800	3052150	0.06	0.011	0.5	F, B	Mesotrophic
Midge	597700	3054150	0.27	0.054	8.2	F	Ultra-oligotrophic
Chester	597500	3053550	0.09	0.039	5.0	F	Ultra-oligotrophic
Limnopolar	597100	3052200	0.58	0.023	5.5	F, B	Oligotrophic

**Table 2:** Statistical and diversity parameters derived from the FAPROTAX and Biolog Ecoplates analyses. C:N:S ratios stand for the ratios among the number of activities related to the C, N, and S cycles in each sample. For the lake samples, S stands for surface samples, and D stands for deep (bottom) samples.

		Refugio S	Turbio S	Escondido S	Somero S	Midge S	Chester S	Chester D	Limnopolar S	Limnopolar D
FAPROTAX	Chao-1	20	8	15	6	15	12	14	8	9
	Shannon *H*	2.00	1.64	1.53	0.75	1.75	1.55	1.97	1.41	1.56
	Evenness *J*	0.67	0.790	0.56	0.42	0.65	0.62	0.750	0.68	0.71
	Menhinick	1.07	0.22	0.43	0.17	0.52	0.47	0.40	0.240	0.26
	C:N:S ratio	3:0.9:1	60:41:1	65:6:1	15:0.8:1	18:4:1	8:3:1	3:0.4:1	30:10:1	3:1:1
BIOLOG	Richness *S*	25	-	-	23	-	-	-	23	23
	Shannon *H*	3.12	-	-	3.09	-	-	-	2.93	2.89
	Evenness *J*	0.97	-	-	0.99	-	-	-	0.93	0.92
	*AWCD*	1.32	-	-	1.39	-	-	-	0.71	0.83

## Data Availability

Illumina sequences are available in the Sequence Read Archive (SRA) of the National Center for Biotechnology Information (NCBI), BioProject accession number PRJNA528697.
